# Bladder cancer in HIV-infected adults: an emerging concern?

**DOI:** 10.7448/IAS.17.4.19647

**Published:** 2014-11-02

**Authors:** Sylvain Chawki, Guillaume Ploussard, Claire Montlahuc, Jérome Verine, Pierre Mongiat-Artus, François Desgrandchamps, Jean-Michel Molina

**Affiliations:** 1 Infectious Diseases, Saint Louis Hospital, Paris, France; 2 Urology, Saint Louis Hospital, Paris, France; 3 Biostatistic and Medical Information, Saint Louis Hospital, Paris, France; 4 Pathology, Saint Louis Hospital, Paris, France

## Abstract

**Introduction:**

As HIV-infected patients get older more non-AIDS-related malignancies are to be seen. Cancer now represents almost one third of all causes of deaths among HIV-infected patients [1]. Albeit bladder cancer is one of the most common malignancy worldwide [2], only 13 cases of bladder cancer in HIV-infected patients have been reported in the literature so far [3].

**Materials and Methods:**

We conducted a monocentric study in our hospital. We selected all patients who were previously admitted (from 1998 to 2013) in our hospital with diagnoses of HIV and bladder cancer. The objective was to assess the prevalence and characteristics of bladder cancers in HIV-infected patients in our hospital.

**Results:**

Based on our administrative HIV database (6353 patients), we found 15 patients (0.2%) with a bladder cancer. Patients’ characteristics are presented in Table 1. Patients were mostly men and heavy smokers. Their median nadir CD4 cell count was below 200 and most had a diagnosis of AIDS. A median time of 14 years was observed in those patients, between the diagnosis of HIV-infection and the occurrence of bladder cancer, although in patients much younger (median age 56) than those developing bladder cancer without HIV infection (71.1 years) [4]. Haematuria was the most frequent diagnosis circumstance in HIV-infected patients who had relatively preserved immune function on highly active antiretroviral therapy (HAART). Histopathology showed relatively advanced cancers at diagnosis with a high percentage of non transitional cell carcinoma (TCC) tumor and of TCC with squamous differentiation, suggesting a potential role for human papilloma virus (HPV) co-infection. Death rate was high in this population.

**Conclusions:**

Bladder cancers in HIV-infected patients remain rare but occur in relatively young HIV-infected patients with a low CD4 nadir, presenting with haematuria, most of them being smokers, and have aggressive pathological features that are associated with severe outcomes.

**Table 1:** Characteristics

Parameters	Per cent or median [IQR]
No. of patients	15
**Patients**	
Age at the diagnosis of HIV-infection (years)	42 [34;47]
Age at the diagnosis of cancer (years)	56 [47;61]
Male gender	73.3%
Smokers (and former smokers)	73.3%
**HIV infection**	
Nadir CD4 cell count (cells/mm^3^)	195 [95;262]
CD4 cell count (cells/mm^3^)	506 [228;703]
CDC stage C at diagnosis	54.6%
% with plasma HIV RNA level < 200 cp/mL	60.0%
% on HAART at diagnosis	78.6%
**Bladder cancer**	
*Histological type*	
Transitional cell carcinoma	80.0%
*Including urothelial carcinoma with squamous differentiation*	*27.3%*
Sarcomatoid carcinoma	13.3%
Epidermoid carcinoma	6.7%
*T stage of the tumor*	
a	26.7%
1	26.7%
2	46.6%
High histological grade of tumor	69.2%
Haematuria as initial symptom	71.4%
Death due to bladder cancer	30.8%
